# Shengmai injection inhibits palmitic acid-induced myocardial cell inflammatory death via regulating NLRP3 inflammasome activation

**DOI:** 10.1016/j.heliyon.2023.e21522

**Published:** 2023-11-02

**Authors:** Gang Yin, Zi-qing Hu, Jing-ya Li, Zhong-yu Wen, Yong-qin Du, Peng Zhou, Liang Wang

**Affiliations:** a Department of Integrated Traditional Chinese and Western Medicine, Anhui University of Chinese Medicine, Hefei, Anhui, 230012, China; b Research Institute of Integrated Traditional Chinese and Western Medicine, Anhui Academy of Chinese Medicine, Hefei, Anhui, 230012, China

**Keywords:** Shengmai injection, NLRP3/Caspase-1 signaling pathway, Diabetic cardiomyopathy, Pyroptosis

## Abstract

**Objective:**

To determine the protective effect of Shengmai injection (SMI) on myocardial injury in diabetic rats and its mechanism based on NLRP3/Caspase1 signaling pathway.

**Materials and methods:**

Rat H9c2 cardiomyocytes were cultured *in vitro*, and the cell survival rate of different concentrations of palmitate acid (PA) and different concentrations of SMI were detected by CCK-8. The myocardial injury cell model was induced with PA, treated with SMI, and combined with NLRP3 specific inhibitor (MCC950) to interfere with the high-fat-induced rat H9c2 myocardial cell injury model. The cell changes were observed by Hoechst/PI staining and the expression levels of MDA, SOD, and ROS in each group were detected. The protein and gene changes of the NLRP3/Caspase-1 signaling pathway were detected by Western blot and RT-qPCR, respectively.

**Results:**

200 μmol/L of PA were selected to induce the myocardial injury cell model and 25 μL/mL of SMI was selected for intervention concentration. SMI could significantly reduce MDA expression, increase SOD level, and decrease ROS production. SMI could decrease the gene expression levels of NLRP3, ASC, Caspase-1, and GSDMD, and the protein expressions of NLRP3, ASC, Cleaved Caspase-1, GSDMD, and GSDMD-N.

**Conclusion:**

SMI can inhibit the high-fat-induced activation of the NLRP3/Caspase-1 signaling pathway, intervene in cardiomyocyte pyroptosis, and prevent diabetic cardiomyopathy.

## 1 Introduction

Diabetes is a metabolic disease characterized by chronic hyperglycemia. At present, with the continuous improvement of people's living standards, the incidence of diabetes is increasing [1]. Diabetes can induce a series of complications, including pathological changes in the heart, blood vessels, kidneys, nerves, and feet [2]. Diabetic cardiomyopathy (DCM) will change the heart function, cause myocardial ischemia and heart failure, and eventually death, which affects the heart function, causing ischemia, and heart failure [3]. Recent studies have shown that abnormal glucose and lipid metabolism of cardiomyocytes, inflammation, oxidative stress and cardiomyocyte apoptosis can lead to the occurrence of DCM [4].

Continuous exposure to high glucose and high fat plays a crucial role in the occurrence and development of DCM [5]. Lipid toxicity can induce the apoptosis of H9c2 cardiomyocytes, leading to impaired cardiac function. Lipotoxicity can also cause pathological changes in blood vessels, kidneys, nerves, and feet [6]. High-fat-induced cardiomyocyte injury may lead to energy metabolism disorder, increase the requirement of free fatty acids in cardiomyocytes, and lead to a series of adverse reactions in cardiomyocytes [7]. Long-term exposure of cells to a high-fat environment will promote the occurrence of oxidative stress, resulting in myocardial cell oxidation and antioxidant dysfunction, and finally induces the apoptosis of cardiomyocytes [8].

Shengmai injection (SMI), composed of Ginseng, Radix Ophiopogonis, and Schisandra Chinensis, has therapeutic effects on myocardial ischemia-reperfusion, coronary heart disease, chronic heart failure, and other heart diseases, such as doxorubicin-induced cardiotoxicity [[9], [10], [11], [12]]. SMI alleviated myocardial injury in diabetic rats by activating AMPKα and inhibiting oxidative stress injury mediated by NADPH oxidase (NOX) [13]. The common causes of the development of diabetes, such as the disorder of glucose and lipid metabolism, have been proven to induce NLRP3 overactivation, which is a key factor in inducing pyroptosis [14]. NLRP3 inflammasome induced caspase-1 activation can lead to pyroptosis of myocardial cells, which is the cause of the progression of myocardial dysfunction and the occurrence and development of DCM [15]. Our previous study found that Shengmai Yin (SMY) can reduce the occurrence and development of DCM by regulating the NLRP3/caspase-1 signaling pathway [16], however, it is not clear whether SMI alleviates high-fat-induced myocardial cell injury by regulating NLRP3 signaling pathway.

High concentrations of palmitic acid (PA) have been shown to increase cell death in cultured cardiomyocytes in the development of obesity and DCM [17]. Therefore, *in vitro* models of lipotoxicity of H9c2 cardiomyocytes induced by PA were used in this study. H9c2 cardiomyocytes were taken as the research object, and the pyroptosis of cardiomyocytes was induced by PA to establish an *in vitro* model, to determine the mechanism of SMI in preventing PA-induced pyroptosis by inhibiting NLRP3 overactivation.

## 2 Materials and methods

### 2.1 Materials

Shengmai Injection (20082201) was purchased from Yisheng Pharmaceutical Co., LTD (Jilin, China). MCC950 (HY-12815A, C_20_H_23_N_2_NaO_5_S; Molecular weight: 426.46) from MCE Inc (USA). Palmitic acid (C_16_H_32_O_2_, AS321104) was purchased from BIOBOMEI Corporation. Albumin bovine serum albumin V (non-fatty acid) (CS9070) was purchased from G-CLONE Biotechnology Co., LTD (Tianjin, China). Dulbecco's modified eagle medium high glucose (BL304A), trypsin-EDTA solution (BL501A) and fetal bovine serum (BL201A) were purchased from Labgic Technology Co., Ltd (Beijing, China). Penicillin-streptomycin solution (CM0001) and CCK-8 solution (CT0001) were purchased from SparkJade Biotechnology Co., Ltd (Shandong, China). Reverse transcription kit (BL696A) was purchased from Biosharp. RNA extract (G3013), SYBR Green (G3326-05) and Hoechst33342 solution (G1127) were purchased from Wuhan Servicebio Technology Co., Ltd (Wuhan, China). Propidium iodide PI solution (1 mg/mL) (BL807A) and ROS detection kit (BL714A) were purchased from Biosharp.

### 2.2 Cell culture

H9c2 cell (CL-0089) was purchased from Wuhan Procell Life Technology Co., LTD, cultured in high-glucose medium containing 10 % fetal bovine serum in an incubator containing 5 % CO_2_ at 37 °C.

### 2.3 Experimental grouping

The experimental groups are as follows: control group (CTL), palmitic acid group (PA), SMI group (SMI), NLRP3 inhibitor group (MCC950, 10 μmol/L), SMI + NLRP3 inhibitor group (SMI + MCC950).

### 2.4 Detection of different concentrations of PA on H9c2 cell viability

0.0307 g PA was added into 3 mL sodium hydroxide solution with a concentration of 0.1 mol/L and fully saponified in 75 °C water bath for 30 min. 40 % BSA solution without short-chain fatty acids was configured and evenly mixed with PA sodium saponification solution in a ratio of 1:1 to obtain 20 mM PA storage solution. The PA solution was diluted to 100, 200, 400, 800, and 1600 μmol/L 1 × 10^4^ cells per well were planted in 96-well plates, after overnight culture, the cells were randomly divided into 6 groups, namely the control group and PA group (100, 200, 400, 800, 1600 μmol/L). After discarding the supernatant, 100 μL of the configured PA solution of each group was added to the corresponding 96-well plate and incubated for 24 h. A complete culture containing 10 % CCK-8 solution was pre-configured. The solution was sucked into 96-well plates and 100 μL solution was inhaled into each well. The solution was placed in an incubator for 1 h away from light, and OD values were measured and read at 450 nm.

### 2.5 Detection of different concentrations of SMI on H9c2 cell viability

6 × 10^3^ H9c2 cells were planted in each well, and each group was designed with 6 multiple wells. H9c2 cells were randomly divided into 9 groups, namely the control group and SMI group (final concentration of SMI 0.04, 0.2, 1, 5, 10, 25, 50, and 100 μL/mL). After discarding the supernatant, 100 μL of the configured Shengmai injection solution of each group was added to the corresponding 96-well plate and incubated in the incubator for 24 h. A complete culture containing 10 % CCK-8 solution was pre-configured. The solution was sucked into 96-well plates and 100 μL solution was inhaled into each well. The solution was placed in an incubator for 1 h away from light, and OD values were measured and read at 450 nm.

### 2.6 Detection of the levels of MDA and SOD

Cell supernatant of each group was collected, 5000 r/min, centrifuged at 4 °C for 20 min, and the supernatant was used according to the kit instructions.

### 2.7 Detection of ROS

H9c2 cardiomyocytes were inoculated with 2 × 10^5^ in 6-well plates and cultured for 24 h. The culture medium was discarded and the cells were cleaned with DMEM without fetal bovine serum 3 times. The ROS kit reagents were prepared with the serum-free medium at a ratio of 1:1000. 1 mL solution was added to each well and incubated at 37 °C for 30 min. After the discarded solution, 1 mL serum-free medium was added to wash cells 3 times, and the cells were observed and photographed under an inverted fluorescence microscope.

### 2.8 Detection of pyroptosis by Hoechst/PI staining

H9c2 cardiomyocytes were inoculated in 6-well plates at 2 × 10^5^, cultured for 24 h, and washed twice with PBS. Then, 1 mL of Hoechst33342 working solution was added to each well and incubated for 10 min at 37 °C. After dropping the Hoechst solution, the cells were rinsed twice with PBS, and 1 mL of PI solution was added to each well before incubating at 37 °C for 10 min. The PI solution was discarded, cells were washed with PBS 1–2 times, and the 6-well plates were placed under an inverted fluorescence microscope for observation and photography.

### 2.9 Detection of gene expression by RT-PCR

H9c2 cardiomyocytes were inoculated into 6-well plates at 5 × 10^5^. After treating the cells accordingly for 24 h, RNA was extracted from the cells using RNA extraction solution, and the same amount of RNA was reverse transcribed into cDNA. The cDNA of each group was amplified by SYBR on LightCycler®96 PCR apparatus. The mRNA levels of the genes were detected by 2^−ΔΔCq^ method (Table 1).

**Table 1:** Primers used in RT-qPCR.

Primers	Sequence (5' →3′)	
NLRP3	Forward	5′-GAGCTGGACCTCAGTGACAATGC-3′
	Reverse	5′-AGAACCAATGCGAGATCCTGACAAC-3′
Caspase-1	Forward	5′-GCACAAGACTTCTGACAGTACCTTCC-3′
	Reverse	5′-GCTTGGGCACTTCAATGTGTTCATC-3′
GSDMD	Forward	5′-CAGCAGGCAGCATCCTTGAGTG-3′
	Reverse	5′-CCTCCAGAGCCTTAGTAGCCAGTAG-3′
ASC	Forward	5′-ATGGTTTGCTGGATGCTCTGTATGG-3′
	Reverse	5′-AAGGAACAAGTTCTTGCAGGTCAGG-3′
β-actin	Forward	5′-CCCATCTATGAGGGTTACGC-3′
	Reverse	5′-TTTAATGTCACGCACGATTTC-3′

### 2.10 Detection of protein expression by Western blot

H9c2 cardiomyocytes were inoculated into 6-well plates with 5 × 10^5^ cells. Following treating the cells accordingly for 24 h, total proteins were extracted from the cells using protein lysis solution. The proteins in each group were separated on a polyacrylamide gel and deposited to the PVDF membrane in the same quantity. The membrane was first treated in TBST containing 5 % skim milk powder for 2 h before being incubated with the primary antibody overnight at 37 °C.The ratios of primary antibodies used are as follows: anti-NLRP3 (1:1000, ab263899), anti-ASC (1:1000, DF6304), anti-Cleaved caspase-1 (1:1000, AF4005), anti-GSDMD (1:1000, ab219800), anti-GAPDH (1:5000, 380626), and anti-Tubulin (1:5000, AF7011). After washing the PVDF membrane three times with TBST, the PVDF membrane was incubated with the horseradish peroxidase-coupled secondary antibody at room temperature for 2 h before being photographed by Tanon5200.

### 2.11 Statistical analysis

The data were presented as mean ± standard deviation (SD), and SPSS 23.0 was used for statistical analysis. Student's t-test was used to assess the statistically significant difference between two groups. The Dunnett-ANOVA test was used for comparisons between multiple groups. *P* < 0.05 was considered statistically significant.

## 3 Results

### 3.1 Effects of different concentrations of PA on H9c2 cell viability

**Fig. 1:**
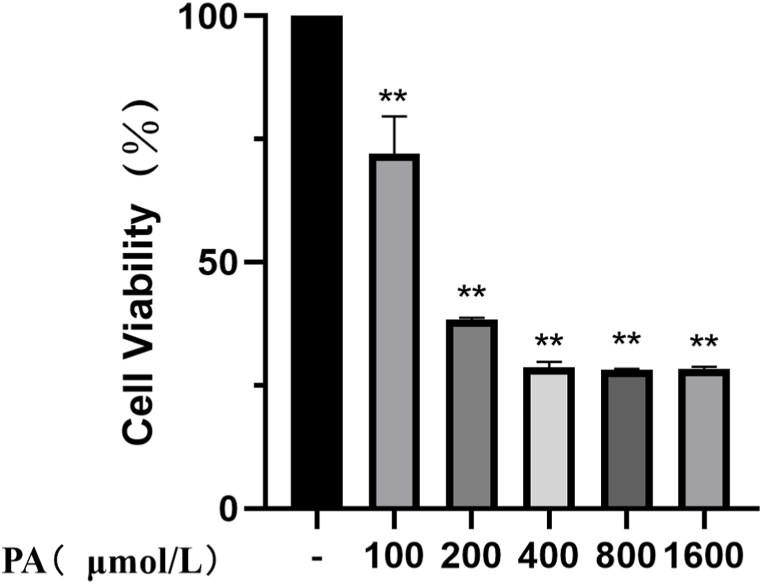
Effect of different concentrations of PA on the viability of H9c2 cells **P < 0.01 vs control group.

The optimal concentration of PA in H9c2 cells was determined by the CCK-8 method. As shown in Fig. 1, compared with the control group, the cell viability of H9c2 decreased to 38.3 % (*P* < 0.05) after intervention with 200 μmol/L of PA. 200 μmol/L PA was selected as the modeling concentration.

### 3.2 Effects of different concentrations of SMI on H9c2 cell viability

**Fig. 2:**
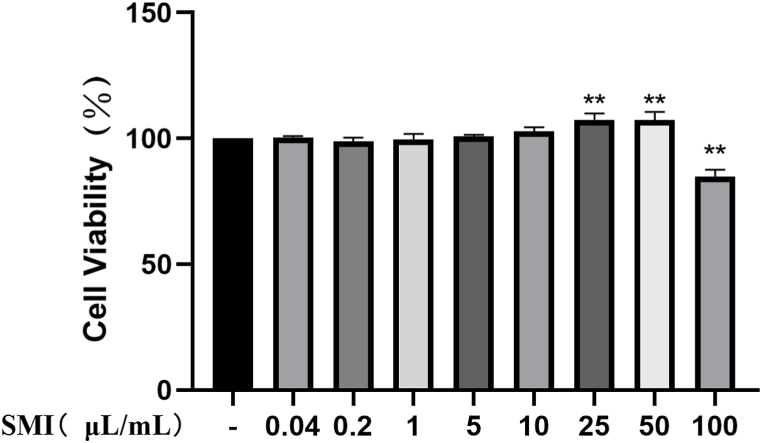
Effect of different concentrations of SMI on viability of H9c2 cells **P < 0.01 vs control group.

Compared with the control group, when the concentration of SMI reached 100 μL/mL, the viability of H9c2 cells was significantly impaired (*P* < 0.05), suggesting that 100 μL/mL was toxic to cells. 25 μL/mL will be selected for subsequent study (Fig. 2).

### 3.3 Effect of SMI on MDA and SOD levels in H9c2 cells

**Fig. 3:**
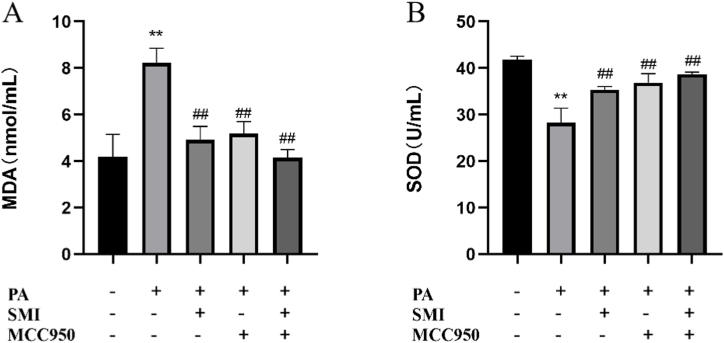
Effect of SMI on MDA and SOD levels in H9c2 cells in each group. (A) The MDA expression levels in H9c2 cells. (B) The SOD expression levels in H9c2 cells. **P < 0.01 vs control group; ^##^*P* < 0.01 vs PA group.

Compared with the control group, MDA level was significantly increased, while SOD level in PA group was significantly decreased (*P* < 0.05). Compared with PA group, the levels of MDA and SOD in SMI group, MCC950 group and SMI + MCC950 group were reversed (*P* < 0.05) (Fig. 3). The results indicated that SMI could significantly improve the oxidative stress level of H9c2 cells induced by PA by inhibiting NLRP3.

### 3.4 Effect of SMI on ROS level in H9c2 cells

**Fig. 4:**
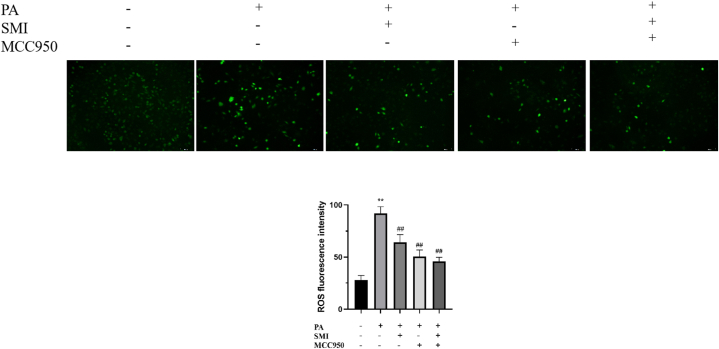
Effect of SMI on ROS level in H9c2 cells in each group (100 × ). **P < 0.01 vs control group; ^##^*P* < 0.01 vs PA group.

ROS levels in H9c2 cardiomyocytes were detected by ROS kit, as shown in Fig. 4. Compared with the control group, PA induction significantly increased ROS production in H9c2, while ROS production was significantly inhibited after SMI and MCC950 treatment.

### 3.5 Effect of SMI on pyroptosis in H9c2 cells

**Fig. 5:**
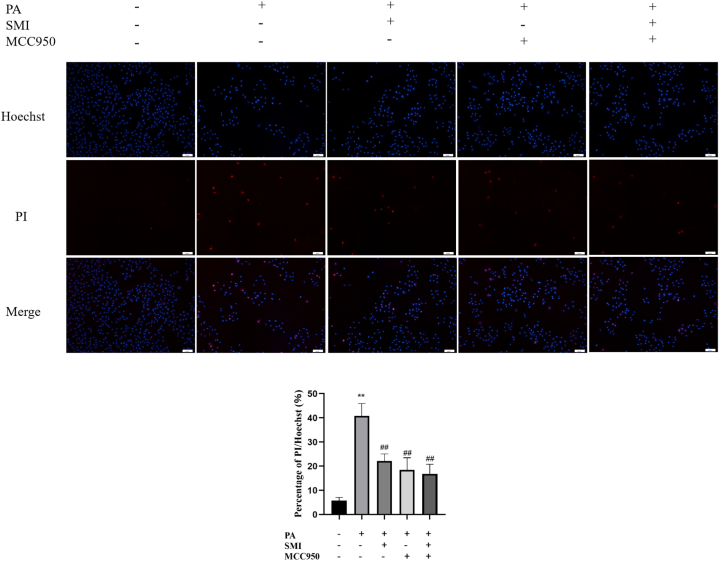
Effect of SMI on pyroptosis in H9c2 cells (100 × ) **P < 0.01 vs control group; ^##^*P* < 0.01 vs PA group.

Hoechst/PI staining was used to detect positive pyroptosis cells, as shown in Fig. 5. Compared with the control group, pyroptosis of H9c2 cells was significantly enhanced by PA, while pyroptosis was significantly inhibited by SMI and MCC950 treatment.

### 3.6 The effect of SMI on the level of genes related to PA-induced pyroptosis

**Fig. 6:**
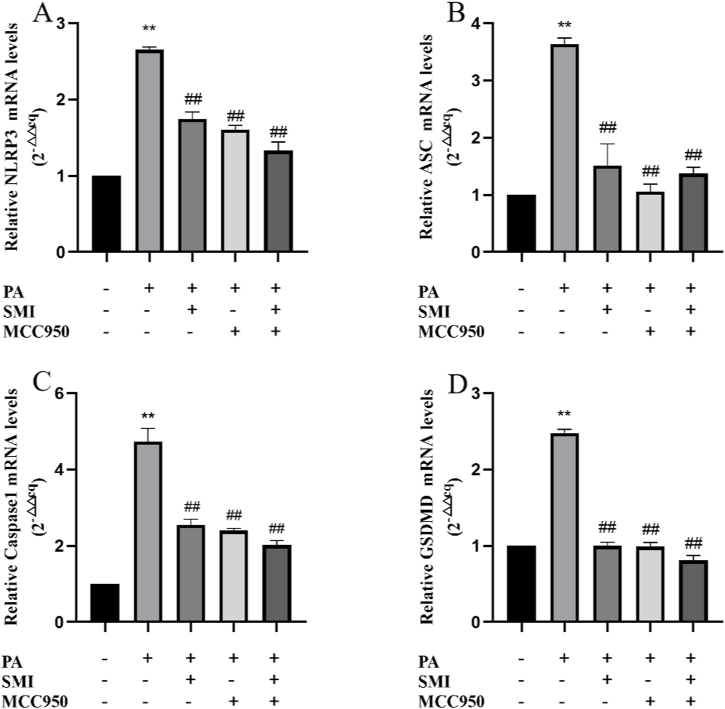
The effect of SMI on the level of genes related to PA-induced pyroptosis. (A) The mRNA expression of NLRP3 in H9c2 cells. (B) The mRNA expression of ASC in H9c2 cells. (C) The mRNA expression of Caspase1 in H9c2 cells. (D) The mRNA expression of GSDMD in H9c2 cells. **P < 0.01 vs control group; ^##^*P* < 0.01 vs PA group.

Compared with the control group, the gene expressions of NLRP3, Caspase1, ASC and GSDMD in PA group were significantly increased (*P* < 0.01). Compared with PA group, gene expression levels of NLRP3 (Fig. 6A), ASC (Fig. 6B), Caspase1 (Fig. 6C) and GSDMD (Fig. 6D) in SMI group, MCC950 group and SMI + MCC950 group were decreased (*P* < 0.01). Therefore, SMI can significantly inhibit PA-induced NLRP3/Caspase1 signaling pathway overactivation.

### 3.7 The effect of SMI on the level of proteins related to PA-induced pyroptosis

**Fig. 7:**
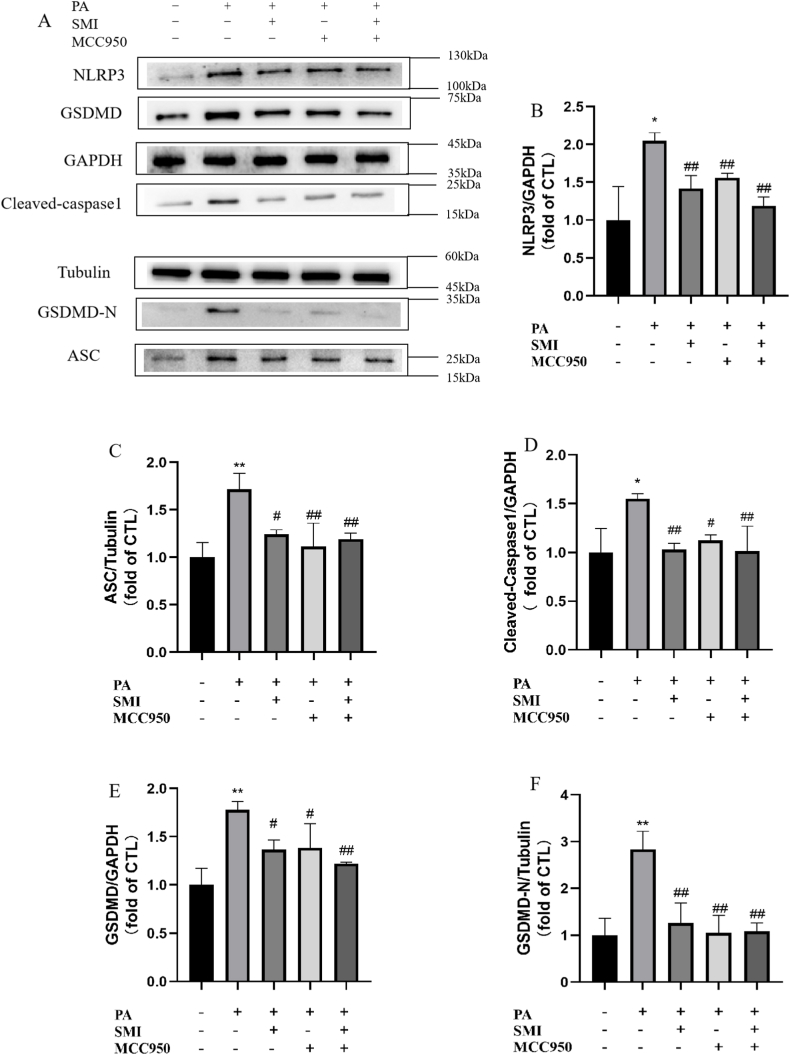
Effect of SMI on the protein expression of the NLRP3/Caspase1 signaling pathway in PA-induced H9c2 cells. (A) The protein expression of NLRP3, GSDMD, Cleaved-caspase1, GSDMD-N and ASC in H9c2 cells. (B) The protein expression of NLRP3 in H9c2 cells. (C) The protein expression of ASC in H9c2 cells. (D) The protein expression of Cleaved-Caspase1 in H9c2 cells. (E) The protein expression of GSDMD in H9c2 cells. (F) The protein expression of GSDMD-N in H9c2 cells. **P < 0.01 vs control group; ^#^*P* < 0.05, ^##^*P* < 0.01 vs PA group.

Compared with the control group, the protein expressions of NLRP3 (Fig. 7A and B), ASC (Fig. 7A and C), Cleaved Caspase1 (Fig. 7A and D), GSDMD (Fig. 7A and E), and GSDMD-N (Fig. 7A and F) were significantly increased (*P* < 0.05, *P* < 0.01), suggesting that hyperlipemia-induced pyroptosis in H9c2 cardiomyocytes. However, the levels of pyroptosis-related proteins in the SMI group, MCC950 group and SMI + MCC950 group were significantly reduced (P < 0.05, P < 0.01).

## 4 Discussion

In T2DM, myocardial lipid metabolism dysfunction is a major cause of myocardial fibrosis and inflammation. T2DM can result in hyperglycemia, hyperinsulinemia, and insulin resistance. These pathogenic alterations result in decreased glucose consumption and increased availability of fatty acids [18]. While the heart is not a primary lipid storage organ, hyperlipidemia in diabetes and obese patients causes an excess of fatty acids in cardiomyocytes, which are converted to triglycerides and deposited in cardiomyocytes [19]. Excessive accumulation of lipids can lead to the production of lipid toxic intermediates in the heart, which eventually lead to inflammation, and myocardial fibrosis [20]. Pathologically, ATP is acquired by cardiomyocytes through β oxidation of free fatty acids (FFA) caused by insulin resistance or insulin deficiency [21]. Cardiolipid toxicity produced by excessive FFA buildup and abnormal β oxidation in cardiomyocytes is a major contributor to the formation and progression of DCM [22,23].

As lipid metabolism disorder is one of the important factors mediating the occurrence and development of diabetic cardiomyopathy [24,25]. Previous animal experiments have confirmed that the blood lipid level of diabetic model rats is significantly increased, and Shengmai injection could ameliorate the elevated blood lipid level in model rats. Combined with in vivo experiments that have confirmed the existence of pyroptosis in DCM rats, palmitic acid (PA) is often used as an *in vitro* model of lipid toxicity induced by H9c2 cardiomyocytes [[26], [27], [28]]. Previous studies have confirmed that the high glucose concentration in the conventional study range is not enough to activate pyroptosis, and it has been proved that 50 mmol/L of high-glucose solution can induce pyroptosis of cells, but the use of high-concentration high-glucose solution to treat cells will cause hypertonic effects on cells [29,30]. Therefore, high-fat induced cardiomyocytes were chosen in this experiment.

PA is a free saturated fatty acid that is prevalent in human plasma. High concentrations of PA have been shown to increase cell death in cultured cardiomyocytes, and its induced myocardial damage is considered to be a key factor in the development of obesity and DCM [31,32]. Therefore, inhibition of lipid toxicity is an important way to delay the deterioration of DCM. Numerous studies have shown that PA is closely related to the activation of NLRP3 inflammasome. PA activates NLRP3 inflammatories before inducing an inflammatory response in sebum cells [33], temporary high-level exposure to PA during pregnancy in mice activates NLRP3 inflammasome and causes placental inflammation [34], and PA can cause the activation of NLRP3 inflammasome, which can damage myocardial cells [35]. Therefore, PA was selected to construct *in vitro* in H9c2 cells.

ROS is mainly produced in mitochondria through normal cell metabolism and is an important molecule mediating normal physiological signal transduction [36]. Studies have confirmed that PA can promote ROS production in different types of cardiomyocytes [37]. The results showed that 200 μmol/L PA induced the increase of ROS in H9c2 cardiomyocytes, and the levels of MDA and SOD were significantly changed, suggesting that PA induced ROS production in H9c2 cardiomyocytes, and the level of oxidative stress was increased. Hoechst/PI staining showed that PA could induce pyroptosis in H9c2 cardiomyocytes. Therefore, PA could induce oxidative stress and pyroptosis in H9c2 cardiomyocytes. A large amount of lipid accumulation in H9c2 cells will lead to aggravate oxidative stress and increase ROS production, which will further activate NLRP3 inflammasome. After activation of NLRP3 inflammasome, caspase 1 can be cut into activated caspase 1, and GSDMD can be divided into GSDMD-N to form membrane pores to induce cell lysis, and ultimately to induce pyroptosis of cardiomyocytes [38,39]. Compared with PA group, ROS levels and pyroptosis levels were significantly inhibited after MCC950 treatment. The results of RT-qPCR and Western blot showed that, compared with the control group, NLRP3, Caspase1, ASC, GSDMD and the related genes and proteins were significantly increased after PA treatment, and MCC950 treatment revised the results. Hence, NLRP3 is a key target for regulating PA-induced myocardial cell damage. SMI can reduce PA-induced increase of ROS and pyroptosis. SMI could significantly inhibit the expression levels of NLRP3 and the related proteins and genes in PA-induced H9c2 cardiomyocytes, which found that SMI can inhibit the NLRP3/Caspase1 signaling pathway in PA-induced H9c2 cardiomyocytes.

## 5 Conclusion

In this study, we found high fat can activate NLRP3/Caspase1 pathway and induce pyroptosis of myocardial cells, which is an important pathological process of DCM. SMI can inhibit hyperlipid-induced pyroptosis of cardiomyocytes by regulating NLRP3/Caspase1 signaling pathway, thus providing a new way to prevent and treat DCM.

## Availability of data and materials

All data used to support the findings of this study are available from the corresponding author upon request.

## Funding statement

This work was supported by 10.13039/501100001809National Natural Science Foundation of China, China (81603602), Key Project Foundation of Natural Science Research in Universities of Anhui Province in China (No. KJ2021A0582), Open Fund Project of Anhui Acupuncture and Moxibustion Clinical Medicine Research Center (No. 2021zjzx09).

## CRediT authorship contribution statement

**Gang Yin:** Funding acquisition, Project administration, Writing – original draft. **Zi-qing Hu:** Data curation, Project administration. **Jing-ya Li:** Project administration, Validation. **Zhong-yu Wen:** Data curation, Formal analysis, Validation. **Yong-qin Du:** Methodology, Validation. **Peng Zhou:** Conceptualization, Writing – review & editing. **Liang Wang:** Conceptualization, Writing – review & editing.

## Declaration of competing interest

The authors declare that they have no known competing financial interests or personal relationships that could have appeared to influence the work reported in this paper.
