# Supplementary material for: Shengmai injection inhibits palmitic acid-induced myocardial cell inflammatory death via regulating NLRP3 inflammasome activation
Source: Heliyon. 2023 Nov 2;9(11):e21522. doi: 10.1016/j.heliyon.2023.e21522 (PMC10660519; doi:10.1016/j.heliyon.2023.e21522)

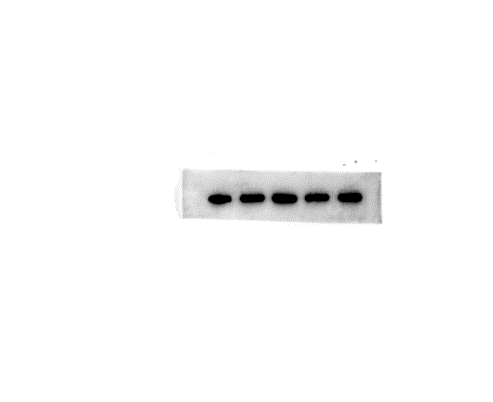

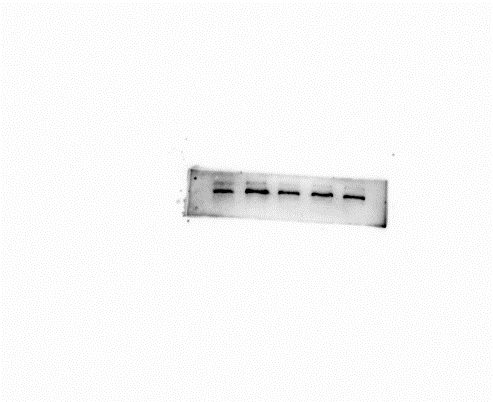
 GAPDH-1 NLRP3-1

GAPDH-2 NLRP3-2


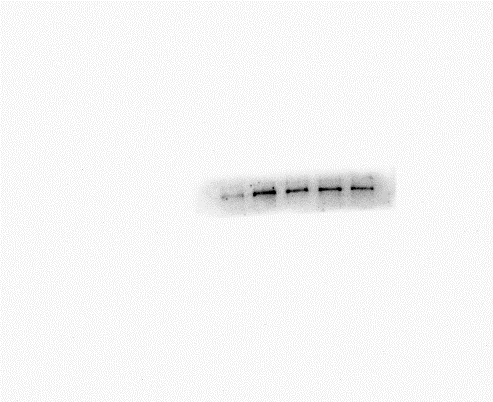

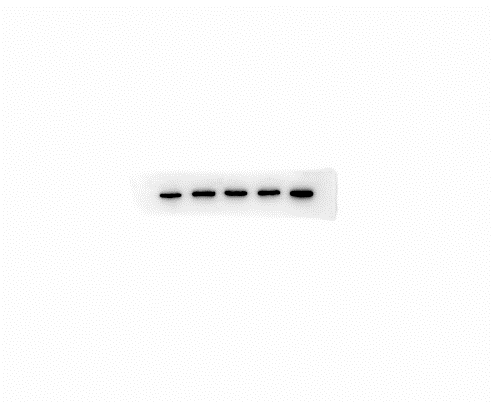


GAPDH-3 NLRP3-3


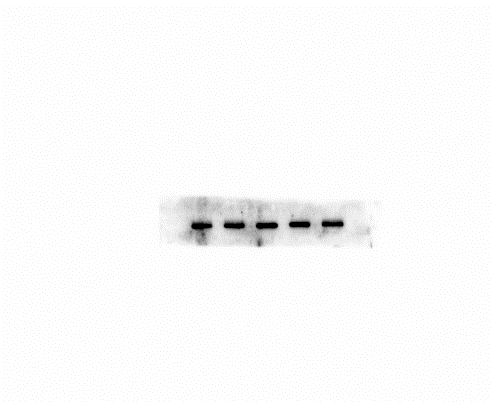

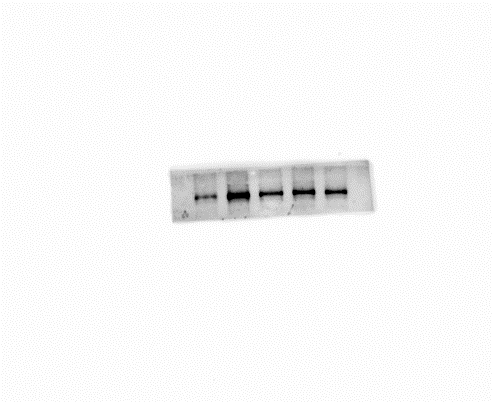


GAPDH-1 GSDMD-1


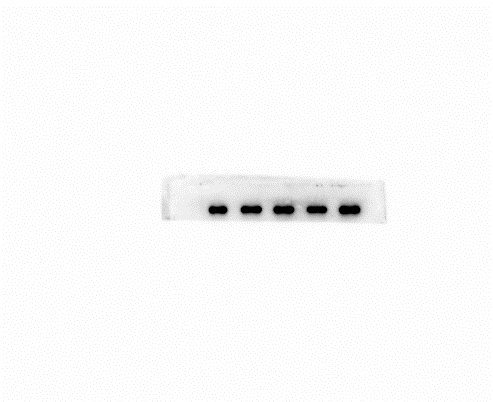


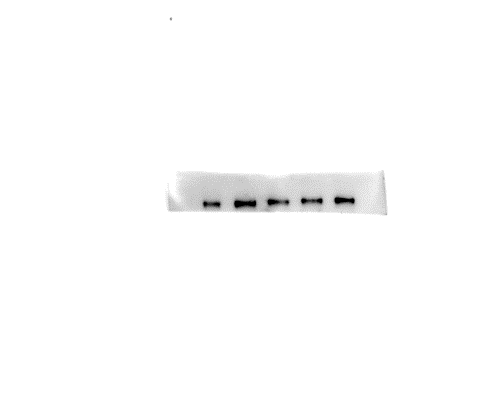


GAPDH-2 GSDMD-2


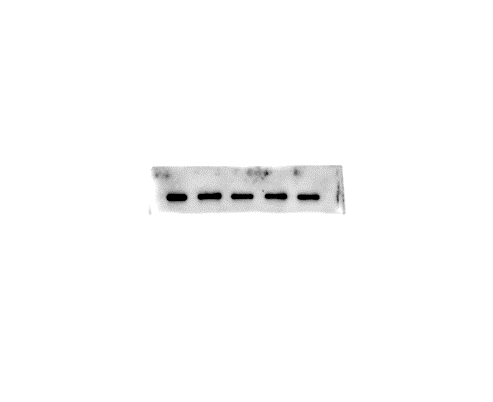

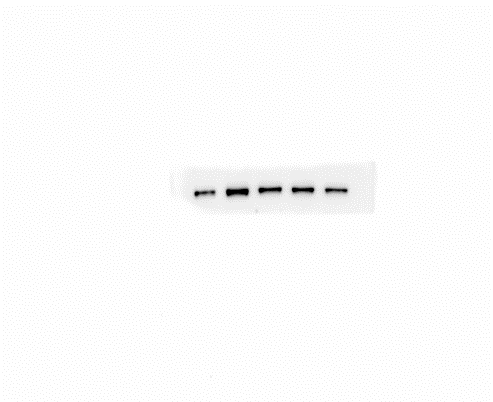


GAPDH-3 GSDMD-3


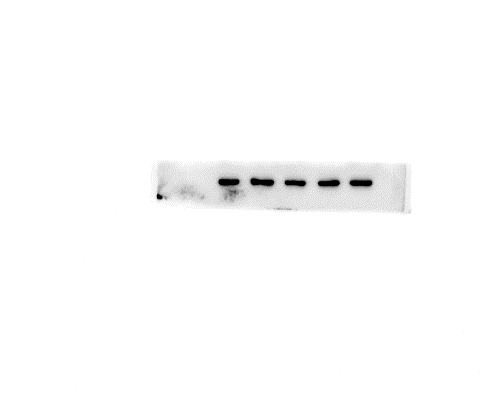

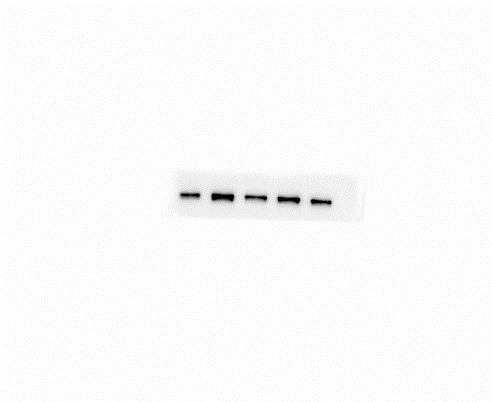


GAPDH-1 Cleaved-caspase1-1


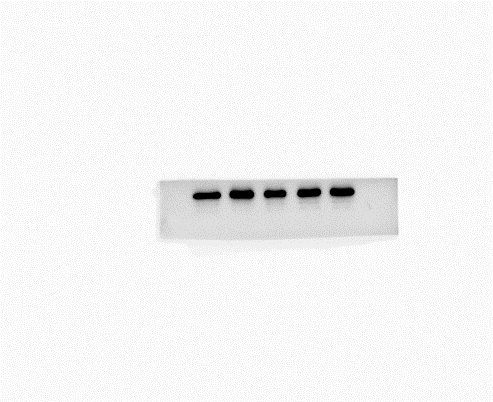

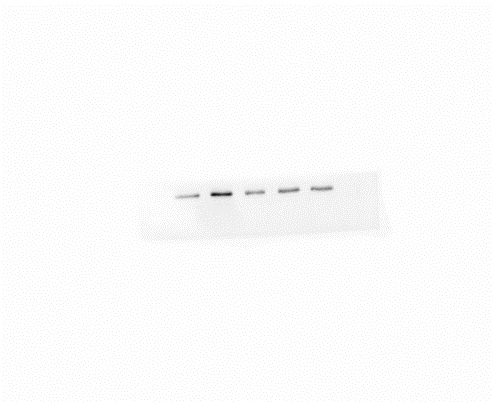


GAPDH-2 Cleaved-caspase1-2


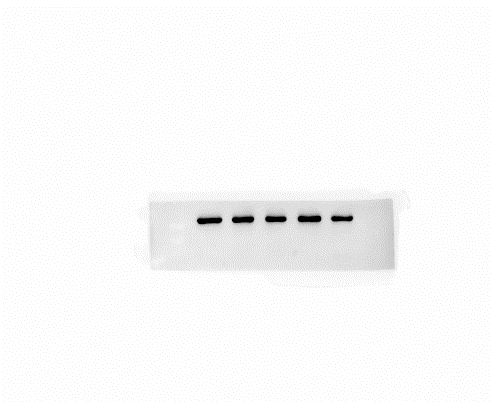

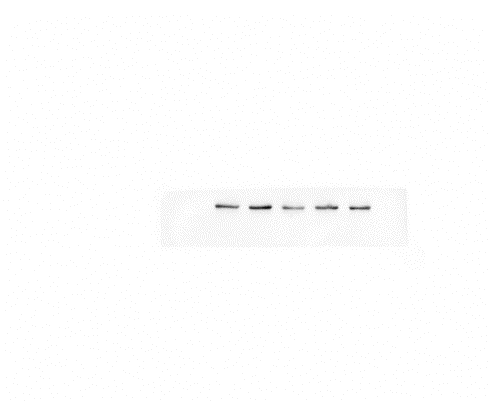


GAPDH-3 Cleaved-caspase1-3


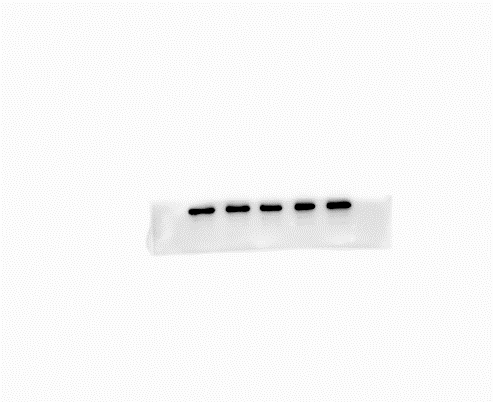

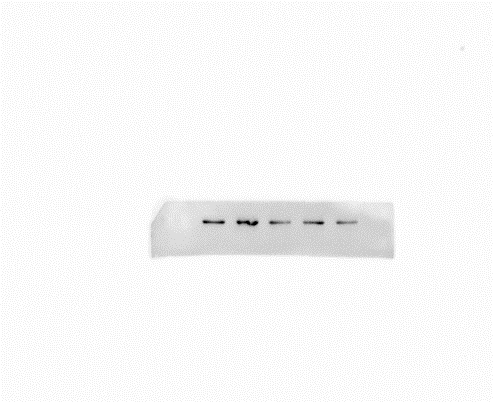


Tubulin-1 ASC-1


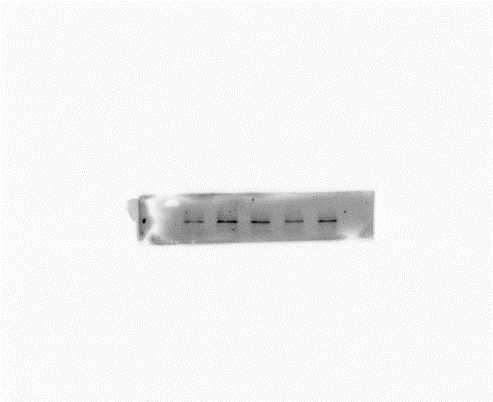


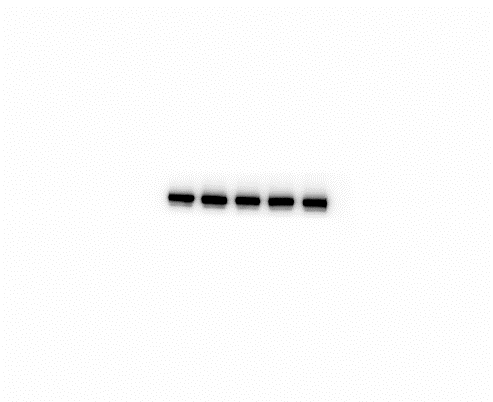


Tubulin-2 ASC-2


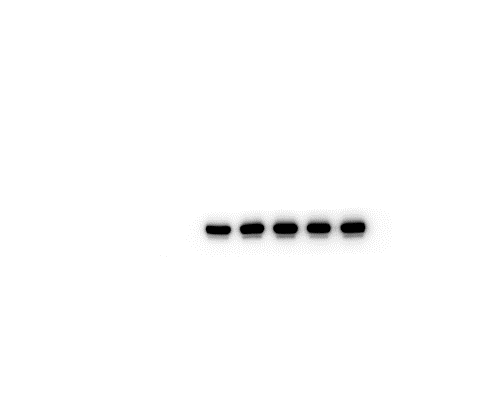

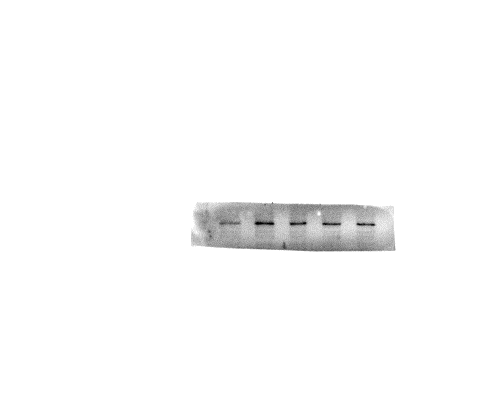


Tubulin-3 ASC-3


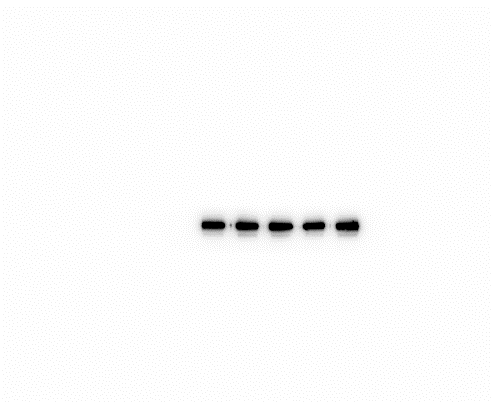

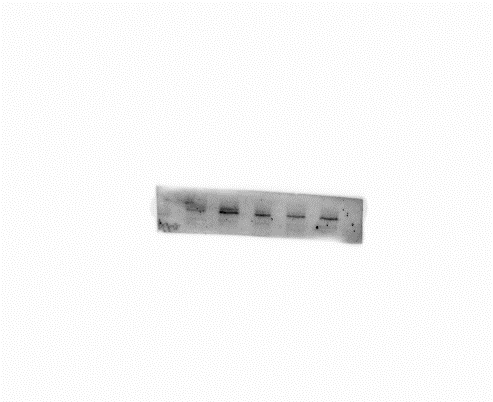


Tubulin-1 GSDMD-N-1


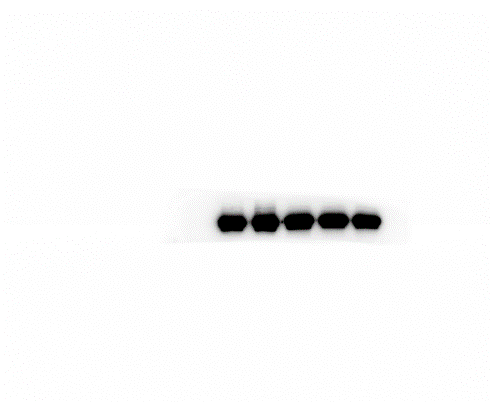

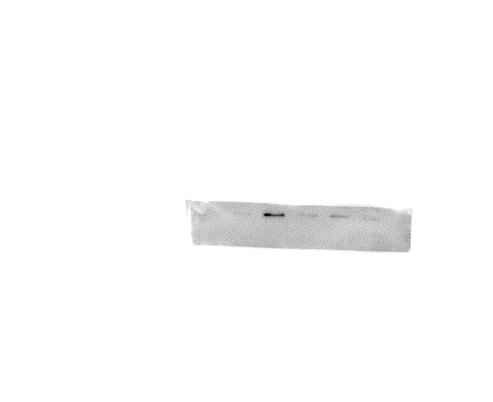


Tubulin-2 GSDMD-N -2


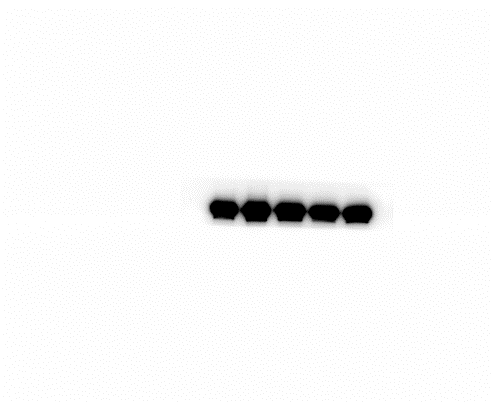

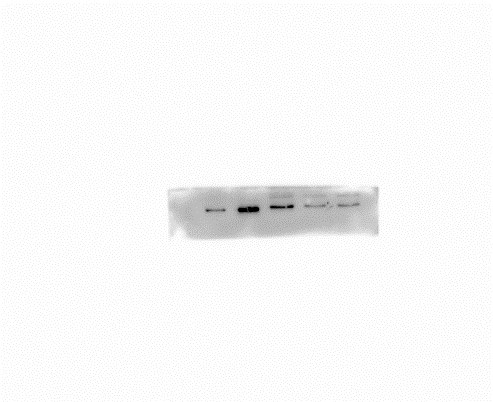


Tubulin-3 GSDMD-N -3


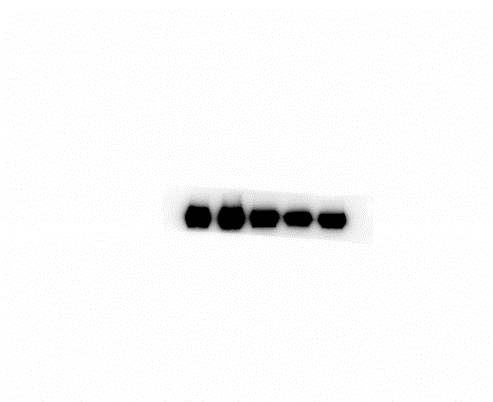

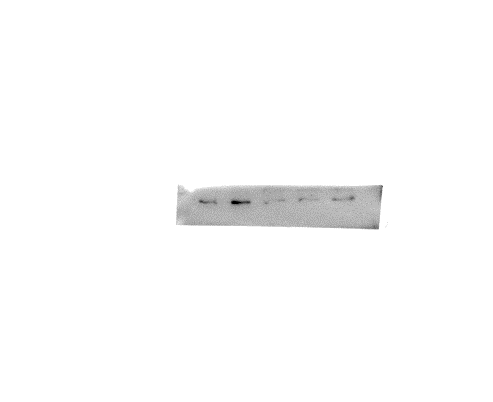

Supplement: Multimedia component 1 [file mmc1.doc]
